# The Dosimetric Impact of Shifts in Patient Positioning during Boron Neutron Capture Therapy for Brain Tumors

**DOI:** 10.1155/2018/5826174

**Published:** 2018-10-01

**Authors:** Jia-Cheng Lee, Yi-Wei Chen, Keh-Shih Chuang, Fang-Yuh Hsu, Fong-In Chou, Shih-Ming Hsu, Sang-Hue Yen, Yuan-Hung Wu

**Affiliations:** ^1^Department of Oncology, Taipei Veterans General Hospital, Taipei, Taiwan; ^2^Department of Biomedical Engineering and Environmental Sciences, National Tsing Hua University, Hsinchu, Taiwan; ^3^School of Medicine, National Yang-Ming University, Taipei, Taiwan; ^4^Nuclear Science and Technology Development Center, National Tsing Hua University, Hsinchu, Taiwan; ^5^Department of Biomedical Imaging and Radiological Sciences, National Yang-Ming University, Taipei, Taiwan; ^6^Institute of Public Health, National Yang-Ming University, Taipei, Taiwan

## Abstract

Unlike conventional photon radiotherapy, sophisticated patient positioning tools are not available for boron neutron capture therapy (BNCT). Thus, BNCT remains vulnerable to setup errors and intra-fractional patient motion. The aim of this study was to estimate the impact of deviations in positioning on the dose administered by BNCT for brain tumors at the Tsing Hua open-pool reactor (THOR). For these studies, a simulated head model was generated based on computed tomography (CT) images of a patient with a brain tumor. A cylindrical brain tumor 3 cm in diameter and 5 cm in length was modeled at distances of 6.5 cm and 2.5 cm from the posterior scalp of this head model (T_6.5 cm_ and T_2.5 cm_, respectively). Radiation doses associated with positioning errors were evaluated for each distance, including left and right shifts, superior and inferior shifts, shifts from the central axis of the beam aperture, and outward shifts from the surface of the beam aperture. Rotational and tilting effects were also evaluated. The dose prescription was 20 Gray-equivalent (Gy-Eq) to 80 % of the tumor. The treatment planning system, NCTPlan, was used to perform dose calculations. The average decreases in mean tumor dose for T_6.5 cm_ for the 1 cm, 2 cm, and 3 cm lateral shifts composed by left, right, superior, and inferior sides, were approximately 1 %, 6 %, and 11 %, respectively, compared to the dose administered to the initial tumor position. The decreases in mean tumor dose for T_6.5 cm_ were approximately 5 %, 11 %, and 15 % for the 1 cm, 2 cm, and 3 cm outward shifts, respectively. For a superficial tumor at T_2.5cm_, no significant decrease in average mean tumor dose was observed following lateral shifts of 1 cm. Rotational and tilting up to 15° did not result in significant difference to the tumor dose. Dose differences to the normal tissues as a result of the shifts in positioning were also minimal. Taken together, these data demonstrate that the mean dose administered to tumors at greater depths is potentially more vulnerable to deviations in positioning, and greater shift distances resulted in reduced mean tumor doses at the THOR. Moreover, these data provide an estimation of dose differences that are caused by setup error or intra-fractional motion during BNCT, and these may facilitate more accurate predictions of actual patient dose in future treatments.

## 1. Introduction

Boron neutron capture therapy (BNCT) is a binary cancer treatment in which compounds containing ^10^B are selectively introduced into tumor cells and then irradiated with thermal neutrons. The ^10^B entities effectively capture thermal neutrons and subsequently emit alpha particles and lithium as shown by the reaction: ^10^B(n, *α*)^7^Li [1]. The track ranges of alpha and lithium particles are only 9 *μ*m and 4 *μ*m, respectively. Thus, BNCT is regarded as a targeted radiotherapy with cell-level accuracy. Because most BNCT research is conducted at nuclear reactors that are not specifically designed for clinical use, patient positioning systems of these reactors remain primitive compared to the linear-accelerators that are installed in hospitals.

In 1996, the Brookhaven Medical Research Reactor (BMRR) group conducted a study on patient positioning [2]. Fiducial marks were placed at anterior, posterior, right and left lateral, and vertex points to facilitate patient positioning and establish patient coordinates. A few years later, patient positioning was more widely addressed again by the BMRR group and additionally by a Finnish group [3, 4].

In 2004, the epithermal neutron beam at the Tsing Hua open-pool reactor (THOR) in Taiwan was renovated [5] and the beam characteristics were subsequently validated [6]. However, setup error in patient positioning remains inevitable. Moreover, for some patients, the ability to maintain a stable supine or sitting position during treatment is very difficult. As a result, patients are likely to exhibit intra-fractional motion during BNCT. The aims of this study were to examine the dosimetric impact of deviations in patient positioning at the THOR where setup-error and intra-fractional patient motion continue to be challenges in the administration of BNCT for brain tumors, and to estimate the resulting dose differences to achieve more accurate predictions of actual patient dose.

## 2. Materials and Methods

### 2.1. Homogeneous Cylindrical Phantom Model

**Figure 1:**
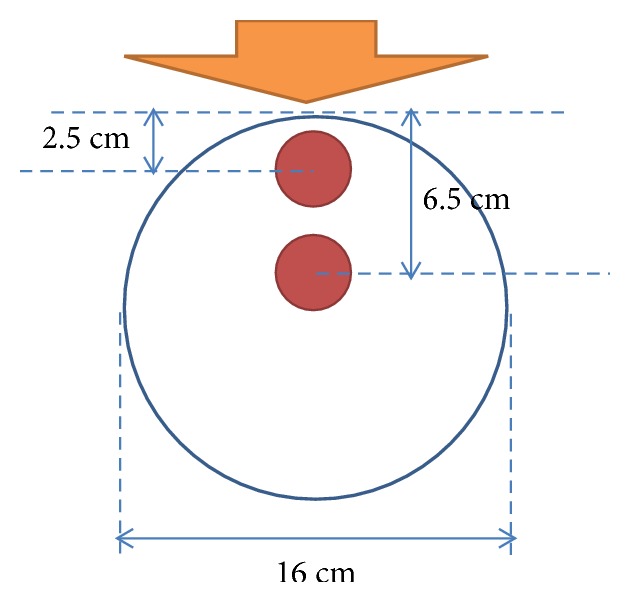
Overview of the cylindrical model established for dose calculations at two different tumor depths. Direction of the neutron beam is indicated with an orange arrow.

To better understand the general dosimetric effects of positioning for a uniform target, a cylindrical polymethyl methacrylate (PMMA) phantom model with a uniform 16 cm diameter was used to simulate the dose applied by irradiation from a neutron source at the THOR (see Figure 1).

### 2.2. Patient Head Model

**Figure 2:**
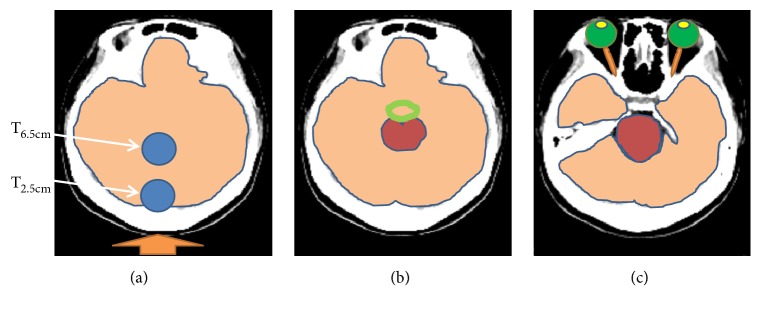
Overview of the simulated head models established for dose calculations at two different tumor depths. Panels (a–c) represent the structures affected by BNCT at tumor depths of 2.5 cm and 6.5 cm. In panel (a), beam direction is indicated with an orange arrow pointing toward the posterior scalp, the blue circles represent the positions of the virtual tumors, and brain tissue is shown in light orange. In panel (b), the circle of Willis is represented as a green circle and the brainstem is shown in dark red. In panel (c), the lenses are shown in yellow, the eyes are shown in green, and the optic nerve is represented by orange lines.

A simulated patient head model was also established with computed tomography (CT) images of a male adult patient with a brain tumor. The central axis of the virtual cylindrical brain tumor was 5 cm in length. The 3 cm width of the tumor was positioned at 6.5 cm and 2.5 cm along the central axis and relative to the posterior scalp (T_6.5cm_ and T_2.5cm_, respectively). It was assumed that the posterior scalp was most proximal to the beam aperture. The beam direction and regions of interest (ROI) in the model included the tumor, normal brain tissue, optic nerve, lenses, eyes, circle of Willis, and brain stem (see Figure 2). Positioning errors were introduced into the model with left and right shifts and superior and inferior shifts of the head model over distances of 1 cm, 2 cm, and 3 cm from the central axis of the beam aperture. In addition, outward shifts from the surface of the beam aperture were made for the model. The effect of rotation was evaluated by a rotation of the patient head model around central axis of the beam aperture. Tilting effect was evaluated by patient head model tilting along the sagittal section of the central axis of the beam. The effects of 5°, 10°, and 15° rotation and tilting were evaluated by averaging both clockwise and counterclockwise. Dose was calculated with the treatment planning system, NCTPlan [7]. The prescribed dose was 20 Gray-equivalent (Gy-Eq) for 80 % of the tumor as previously described in a clinical trial of BNCT for recurrent head and neck cancer that was conducted at the THOR [8]. Beam-on time was determined based on the prescribed tumor dose for the undeviated head model and was consistent among all of the test cases. Material composition was defined according to the International Commission on Radiation Units and Measurements 46 (ICRU-46) report [9].

### 2.3. Neutron Source

An epithermal neutron test beam was constructed for the THOR in 1998 for studies of BNCT. In the summer of 2004, the epithermal beam port for BNCT at this facility was rebuilt [10]. Between 2009 and 2013, the first clinical trial of BNCT for recurrent head and neck cancer was conducted at the THOR [8, 11]. The advantage depth (AD) of the THOR is 8.5 cm [10], assuming a tumor/normal tissue (T/N) ratio of 3.5. The current-to-flux ratio and aperture diameter of the THOR at beam exit are 0.8 and 14 cm, respectively [5]. For the present set of simulated cases, the tumor depth was assumed to be less than the AD for the THOR in order to have a treatment benefit. The current-to-flux ratio of the THOR is 0.8, thereby meeting the recommendation of the International Atomic Energy Agency (IAEA) that this ratio should be greater than 0.7 [12].

### 2.4. Overview of NCTPlan

NCTPlan Ver. 1.1.44 [7] was originally developed by the Harvard/MIT BNCT group and it was used for treatment planning in the present study. Because NCTPlan requires CT images to be formatted as tagged image file format (TIFF) files, ImageJ Ver. 1.48v software [13–15] was used in combination with in-house code to transform the CT images that were collected as Digital Imaging and Communications in Medicine (DICOM) formatted files into TIFF formatted files at 256 × 256 pixel resolution. A total of 125 2 mm thick slices were used and the gray level of the CT images was set to 8 bits. In NCTPlan, the files were further converted into 21 × 21 × 25 voxel images and 56 different materials. Monte Carlo N-Particle Transport Code [16] was used to calculate dose according to F4 tally and kinetic energy release in matter factors.

### 2.5. Dose Calculation Parameters

Dose-rate scaling factor (DRSF) was defined here as a normalization factor that was derived individually for each dose component in the BNCT in-phantom radiation field to provide the best agreement between measured and computed data. The DRSF values derived from measured and computed depth-dose-rate distributions by least-squares criteria were 0.64, 1.39, 0.96, and 0.65, for thermal neutron, fast neutron, photon, and ^10^B, respectively [17]. The T/N ratio for boron concentration in the brain tumor was assumed to be 3.5 according to a previous glioblastoma study [18].

Based on the results of Monte Carlo calculations of relative biological effectiveness (RBE) dose, 3.8 was used for the RBE factor and 1.3 was used for* p*-boron-L-phenylalanine (BPA) in the tumor cells and normal tissue [19]. Previously, an RBE factor of 3.2 was used for THOR epithermal neutron beam high-LET components, such as the products of thermal neutron capture in nitrogen and fast neutrons, while a RBE factor of 0.5 was used for photons [17]. The default neutron power and flux values were 1.2 MW and 1.28 × 10^9^ n·cm^−2^·s^−1^, respectively, for THOR conditions [20]. RBE factors were used to convert physical dose (Gy) to equivalent dose (Gy-Eq). A posterior (180°) field with a 14 cm diameter beam aperture was used. To compare dose distribution and irradiation time among the present cases, the treatment plans were normalized by 20 Gy-Eq to 80 % of the tumor in the absence of position deviation.

### 2.6. Statistical Analysis

All statistical tests were performed with SPSS software (release 17.0, SPSS Inc., Chicago, IL, USA). Two-sided Student's* t-*tests were used to compare dosimetric differences among BNCT plans. Differences with a P-value ≤ 0.05 were considered statistically significant.

## 3. Results

### 3.1. Tumor Dose for the Cylindrical Phantom

Simulated mean tumor dose (D_mean_) and dose to 80 % volume of the tumor (D_80%_) for tumors at depths of 2.5 cm and 6.5 cm (T_2.5cm_ and T_6.5cm_, respectively) from the posterior surface of our cylindrical phantom model were calculated and are summarized in Table 1. Greater shifts in position resulted in greater reductions in applied dose. For example, the average tumor doses for the 1-3 cm left/right lateral shifts and 1-3 cm superior/inferior shifts significantly differed from baseline for the T_6.5cm_ tumor. However, for the T_2.5cm_ superficial tumor, only a lateral shift of 1 cm resulted in a significant difference in average tumor dose from baseline.

### 3.2. Tumor Dose for the Patient Head Model

Greater decreases in D_mean_ and D_80%_ were also associated with greater shifts of the patient head model (Table 2). There are no significant differences in D_mean_ and D_80%_ with a rotation or tilt up to 15° of the patient head model. (Table 3) The average tumor doses for the 1-3 cm left/right lateral shifts and 1-3 cm superior/inferior shifts all significantly differed from baseline. Meanwhile, a 1 cm lateral shift for the T_2.5cm_ superficial tumor and D_80%_ for the T_6.5cm_ tumor was found to significantly differ from baseline as well.

**Figure 3:**
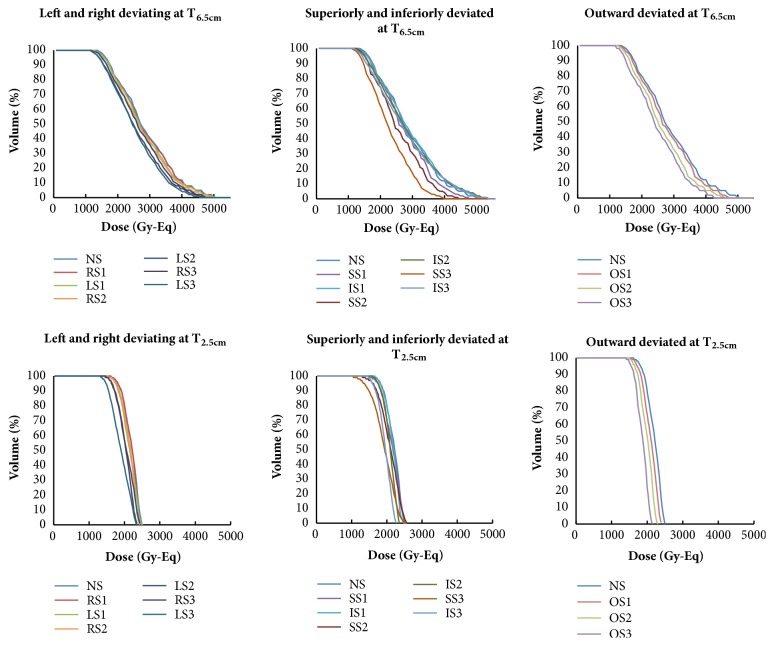
DVHs for T_6.5 cm_ and T_2.5 cm_ tumors that underwent shifts of 1, 2, and 3 cm from their initial positions. NS1: no shift; RS: right shift; LS: left shift; SS: superior shift; IS: inferior shift; OS: outward shift.

Dose-volume histograms (DVHs) of the tumor doses applied to the patient head model are shown in Figure 3. With greater shifts, the dose-volume profiles exhibited broader curves compared with the originally-prescribed dose of 80 % tumor volume with 20 Gy-Eq.

### 3.3. Dose Robustness for Tumors at Different Depths

**Figure 4:**
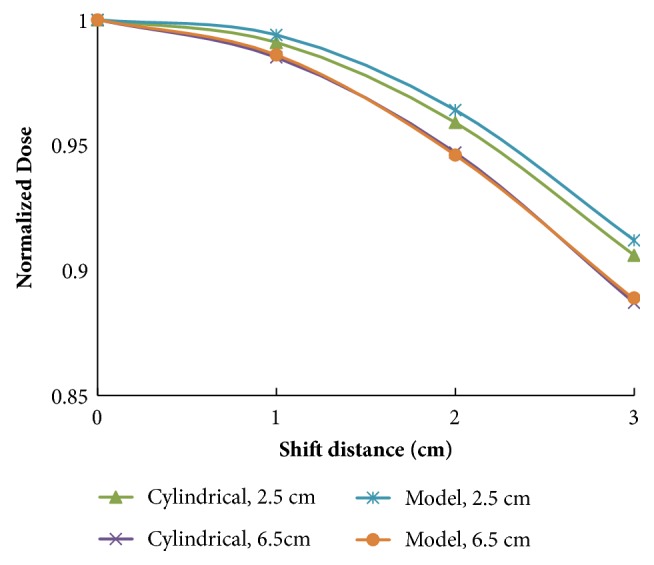
Normalized mean tumor doses for the tumors at 2.5 cm and 6.5 cm in the cylindrical phantom and patient head models that underwent 0–3 cm lateral shifts.

The percentage changes in normalized mean tumor dose for both the cylindrical phantom and patient head models with various shifts are presented in Figure 4. Consistent with the data in Tables 1 and 2, a decline in dose was observed for the T_6.5cm_ tumor when it was shifted and greater decline was associated with the range of lateral shifts compared to the superficial tumor (T_2.5cm_). Thus, tumors at greater depths may be more vulnerable to changes in mean tumor dose with lateral shifts.

### 3.4. Normalized Dose Profile for the Cylindrical Phantom and Patient Head Models

**Figure 5:**
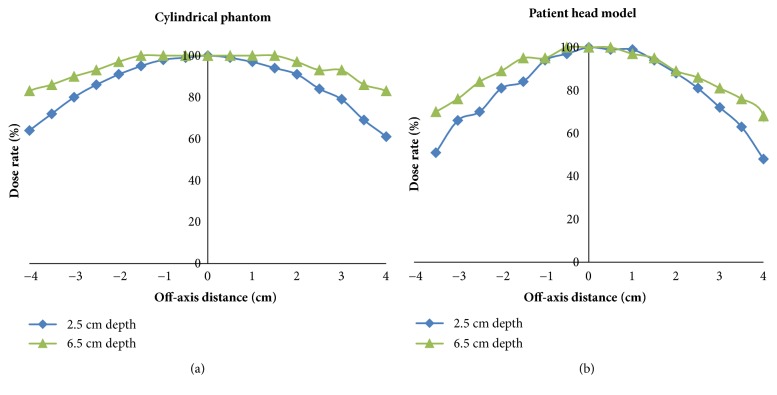
Normalized dose profiles for the (a) cylindrical phantom and (b) patient head models.

Percentage changes in tumor dose rate according to the off-axis distances of the cylindrical phantom and patient head models are shown in Figure 5. Based on these data, it appears that administration of radiation to tumors at a greater depth appears to be less affected by off-axis distances.

### 3.5. Isodose Curves for the Cylindrical Phantom and Patient Head Models with Lateral Shifts

**Figure 6:**
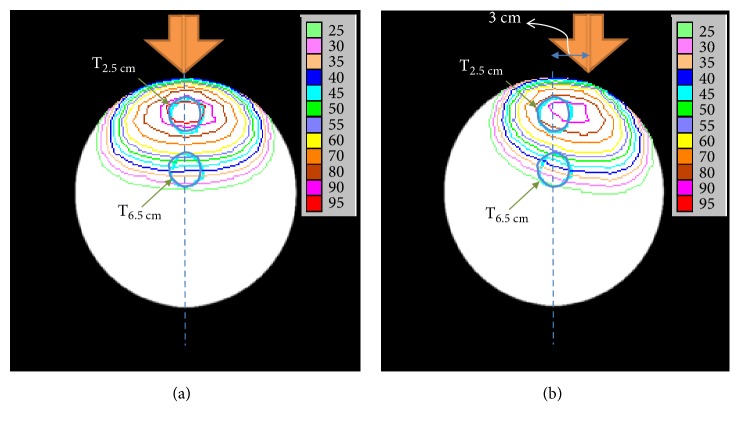
Normalized percentage of isodose curves for the cylindrical phantom model established for dose calculations at two different tumor depths. Panel (a) represents the original position and panel (b) represents a 3 cm lateral shift. The central axis of the neutron beam is indicated with an orange arrow. The positions of the virtual tumors are represented with blue circles.

The effects of lateral shifts on the tumor isodose curves for our cylindrical phantom model are shown in Figure 6. The lateral shifts caused the isodose curves to be shifted and distorted.

### 3.6. Changes in Percent Mean Tumor Dose Compared to BMRR

The effects of positioning shifts on mean tumor doses applied at the THOR and BMRR are compared in Table 4. For our patient head model, the mean tumor doses for T_6.5 cm_ after lateral shifts of 1 cm, 2 cm, and 3 cm were reduced by approximately 1 %, 6 %, and 11 % compared to the dose received at the initial tumor position. The mean tumor doses for T_6.5 cm_ also decreased by approximately 5 %, 11 %, and 15 % for outward shifts of 1 cm, 2 cm, and 3 cm, respectively. Overall, these percent decreases in mean tumor dose were consistently less than those obtained for a tumor model at the BMRR [3].

### 3.7. Normal Tissue Dose with Positioning Shifts

The radiation dose to normal tissues proximal to the T_6.5 cm_ and T_2.5 cm_ tumors with or without shifts of 1 cm, 2 cm, or 3 cm was listed in Table 5. Most of the mean doses to the normal tissues decreased when shifts were introduced into the patient head model. However, only minimal changes in mean dose were observed.

## 4. Discussion

In this study, mean tumor doses decreased following shifts in our cylindrical phantom and patient head models. The greater the shift distance, the greater the decrease in mean tumor dose. The DVHs showed broader curves compared with the originally-prescribed dose. Based on these results, it appears that the mean dose for tumors at greater depths would potentially be more sensitive to deviations in patient positioning. In contrast, the mean dose to normal tissues was relatively unaffected by deviations in positioning.

A study conducted at the BMRR also found that mean tumor dose decreased with larger shifts in patient positioning. For example, in a representative patient with glioblastoma, the mean tumor dose decreased by 2.87 % and 7.19 % with 1.0 cm and 2.0 cm lateral shifts, respectively [3]. The mean tumor doses also decreased by 9.03 % and 15.56 % when an air gap of 1.0 cm and 2.0 cm existed between the patient and the beam port, respectively [2]. However, the effect of tumor depth was not evaluated in this BMRR study.

In another study conducted in Finland, target volume and tumor dose only changed by 1 % for a phantom model that underwent a 0.5 cm displacement in beam position along the perpendicular axis at a depth of 40 mm along the tumor axis compared to its initial position [4]. In addition, the difference in tumor dose for the phantom model at these two positions that corresponded to normal brain doses was less than 5 % along the tumor axis. The effect of a 5 mm displacement on the dose profiles of the center points of an ellipsoidal phantom (8 cm), the target volume (6 cm), and the tumor (4 cm) was also examined. However, the effects of different tumor depths on tumor dose and normal tissue dose were not studied.

To the best of our knowledge, the current study is the first to show that the mean tumor dose for tumors at greater depths could be more vulnerable to positioning error during BNCT. For example, the beam profiles for both the patient model and the cylindrical phantom were more flat for the tumor at the greater depth (T_6.5cm_) in the present study (Figure 5). Moreover, the decrease in proportion of mean tumor dose was greater, in both the cylindrical phantom and the patient head models (Figure 4). The discrepancies between the beam profile and decreased proportion of mean tumor dose may be explained by the isodose curves obtained for both models (Figure 6). These curves show that although positioning deviations existed, the mean tumor dose exhibited a greater decrease for the T_6.5cm_ tumor due to the sharper slope in its isodose curve, thereby resulting in a greater impact with a lateral shift.

Compared to the BMRR study, both our cylindrical phantom and head models, at T_2.5cm_ or T_6.5cm_, had mean tumor doses that were less affected by positioning errors from lateral and outward shifts (Table 4). A report published by the IAEA in 2001 [12] indicated that a forward-directed beam with a current-to-flux ratio greater than 0.7 delivers a higher intensity neutron beam at a distance from the reactor shield face. As a result, greater flexibility has been allowed in patient positioning. The current-to-flux ratio of the THOR at the 14 cm beam aperture surface is 0.8 [5], while the current-to-flux ratio reported for the BMRR at the 12 cm beam aperture surface is 0.67 [19]. The results of the present study from the THOR represent smaller differences than previously observed at the BMRR. However, the latter results derive from an unknown tumor depth of a representative patient. Therefore, the relative robustness of the THOR data may partially be explained by the forwardness of the neutron source.

The mean doses in the left and right lenses, the eyes, and the optic nerve of the simulated model in the present study were only slightly affected by shifts in tumor location. These results may be explained by the distance between these tissues and the beam aperture which extend far beyond the AD of 8.5 cm at the THOR. The mean doses to the brain also did not significantly differ with 1 cm, 2 cm, and 3 cm shifts in T_6.5cm_ and T_2.5cm_. It is possible that the large brain volume of our model accounts for the mean brain dose not being largely unaffected. However, it is predicted that the dose to normal tissues would be greater if the distance between normal tissues and a beam aperture is small.

Based on the results of this study, deviations in tumor dose and normal tissue dose may be estimated for different tumor depths. For example, dose correction factors could be applied according to brain tumor depth to more accurately predict the actual dose that a patient receives during treatment. Furthermore, an increase in treatment time could compensate for the drop-off in radiation dose due to position shifts, while still respecting the constraints for normal tissue. Real-time monitoring techniques involving fiducial markers or surface-guided techniques could also be used to monitor shift distances due to patient motion over an entire treatment time in order to correct the dose that is actually delivered to a patient.

### 4.1. Limitation of the Study

There were limitations associated with this study. First, a tumor model based on a cylindrical phantom or an adult patient with a brain tumor receiving single posterior port irradiation cannot represent all patients. Also, in this study of single-field radiation, the greatest tumor depth modeled was 8 cm. For tumors at depths greater than the AD, better dose distribution may be achieved with multi-field BNCT [21]. Finally, the effects of positioning errors on multifield cases were not evaluated.

## 5. Conclusions

In the present study, greater shift distances resulted in greater decreases in mean tumor dose. The present results also suggest that dose distribution for brain tumors at greater depths receiving BNCT would be more sensitive to positioning deviations. Furthermore, these data provide an estimate of dose differences that may be caused by setup error or intra-fractional motion during BNCT.

## Figures and Tables

**Table 1:** Mean tumor dose (D_mean_) and 80 % dose (D_80%_) with a lateral shift (LS) of the phantom model parallel to the beam exit surface and with an outward shift (OS) with an air gap between the phantom model and the beam exit surface.

**Tumor** **depth & dose**	**Baseline** Gy-Eq	**LS1** Gy-Eq (± SD)	**LS2** Gy-Eq (± SD)	**LS3** Gy-Eq (± SD)	**LS1 *vs.* Baseline** P-value	**LS2 *vs.* Baseline** P-value	**LS3 *vs.* Baseline** P-value	**OS1** Gy-Eq (± SD)	**OS2** Gy-Eq (± SD)	**OS3** Gy-Eq (± SD)
**T**_6.5**cm**_										
**D**_**mean**_	24.31	23.95 (± 0.16)	23.02 (± 0.32)	21.56 (± 0.30)	0.01	< 0.001	< 0.001	22.19	21.43	20.21
**D**_80%_	20.00	19.52 (± 0.14)	18.50 (± 0.53)	17.67 (± 0.24)	< 0.001	0.01	< 0.001	18.25	17.75	16.73

**T**_2.5**cm**_										
**D**_**mean**_	20.69	20.51 (± 0.19)	19.84 (± 0.17)	18.75 (± 0.22)	0.072	0.001	< 0.001	19.81	18.43	17.52
**D**_80%_	20.00	19.85 (± 0.18)	18.66 (± 0.71)	17.67 (± 0.26)	0.053	0.014	< 0.001	18.50	17.79	16.96

D: dose; LS1: 1 cm lateral shift; LS2: 2 cm lateral shift; LS3: 3 cm lateral shift; OS1: 1 cm outward shift; OS2: 2 cm outward shift; OS3: 3 cm outward shift. The mean doses reported for LS1, LS2, and LS3 represent the mean values of the doses for left/right and superior/inferior shifts.

**Table 2:** Mean tumor dose (D_mean_) and 80 % dose (D_80%_) with a lateral shift (LS) of the patient head model parallel to the beam exit surface and with an outward shift (OS) with an air gap between the patient head model and the beam exit surface.

**Tumor** **depth & dose**	**Baseline** Gy-Eq	**LS1** Gy-Eq (± SD)	**LS2** Gy-Eq (± SD)	**LS3** Gy-Eq (± SD)	**LS1 *vs.* Baseline** P-value	**LS2 *vs.* Baseline** P-value	**LS3 *vs.* Baseline** P-value	**OS1** Gy-Eq (± SD)	**OS2** Gy-Eq (± SD)	**OS3** Gy-Eq (± SD)
**T**_6.5**cm**_										
**D**_**mean**_	24.45	24.10 (± 0.23)	23.13 (± 0.34)	21.73 (± 0.24)	0.02	< 0.001	< 0.001	23.30	21.84	20.74
**D**_80%_	20.00	19.92 (± 0.19)	18.72 (± 0.62)	17.84 (± 0.19)	0.22	0.01	< 0.001	19.33	18.34	17.15

**T**_2.5**cm**_										
**D**_**mean**_	20.68	20.55 (± 0.24)	19.93 (± 0.34)	18.85 (± 0.44)	0.17	0.01	0.002	19.70	18.92	18.08
**D**_80%_	20.00	19.89 (± 0.24)	18.74 (± 0.84)	17.83 (± 0.51)	0.12	0.03	0.002	19.11	18.28	17.47

D: dose; LS1: 1 cm lateral shift; LS2: 2 cm lateral shift; LS3: 3 cm lateral shift; OS1: 1 cm outward shift; OS2: 2 cm outward shift; OS3: 3 cm outward shift. The mean doses reported for LS1, LS2, and LS3 represent the mean values of the doses for left/right and superior/inferior shifts.

**Table 3:** Mean tumor dose (D_mean_) and 80 % dose (D_80%_) with a rotation and tilt of the patient head model to the center of beam exit surface.

**Tumor** **depth & dose**	**BL** Gy-Eq	**R5** Gy-Eq (± SD)	**R10** Gy-Eq (± SD)	**R15** Gy-Eq (± SD)	**T5** Gy-Eq (± SD)	**T10** Gy-Eq (± SD)	**T15** Gy-Eq (± SD)	**R5 *vs.* BL** P-value	**R10 *vs.* BL** P-value	**R15 *vs.* BL** P-value	**T5 *vs.* BL** P-value	**T10 *vs.* BL** P-value	**T15 *vs.* BL** P-value
**T**_6.5**cm**_													
**D**_**mean**_	24.45	24.33 (± 0.38)	24.19 (± 0.15)	23.72 (± 0.24)	24.30 (± 0.30)	24.30 (± 0.49)	24.66 (± 0.88)	0.37	0.12	0.07	0.30	0.37	0.40
**D**_80%_	20.00	20.08 (± 0.16)	20.04 (± 0.09)	19.7 (± 0.16)	20.14 (± 0.26)	20.07 (± 0.59)	20.41 (± 0.58)	0.30	0.34	0.12	0.30	0.45	0.25

**T**_2.5**cm**_													
**D**_**mean**_	20.68	20.79 (± 0.15)	20.53 (± 0.07)	20.07 (± 0.19)	20.73 (± 0.07)	20.85 (± 0.14)	20.80 (± 0.26)	0.25	0.10	0.07	0.25	0.17	0.32
**D**_80%_	20.00	20.12 (± 0.13)	19.79 (± 0.14)	19.21 (± 0.33)	19.98 (± 0.06)	19.91 (± 0.18)	19.71 (± 0.30)	0.33	0.11	0.09	0.16	0.23	0.17

BL: baseline; D: dose; R5: 5° rotation; R10: 10° rotation; R15: 15° rotation; T5: 5° tilt; T10: 10° tilt; T15: 15° tilt.

**Table 4:** Percent changes for mean tumor doses at the BMRR and the THOR with lateral shifts of a patient head model parallel to the beam port collimator surface or with an air gap between the patient head model and the beam port due to an outward shift of the head.

Distance and type of shift	BMRR (%) [3]	THOR (%)			
		Model		Cylindrical	
		T_6.5cm_	T_2.5cm_	T_6.5cm_	T_2.5cm_
1 cm lateral shift	2.78	1.43	0.63	1.48	0.87
2 cm lateral shift	7.19	5.52	3.63	5.31	4.11
3 cm lateral shift	-	11.12	8.85	11.31	9.38
1 cm outward shift	9.03	4.70	4.74	4.72	4.25
2 cm outward shift	15.56	10.67	8.51	11.85	10.92
3 cm outward shift	-	15.17	12.57	16.87	15.32

**Table 5:** Doses (± SD) of various normal tissues with tumor depths of 6.5 cm and 2.5 cm and with 0-3 cm head-shifts.

**Organ**		**Baseline**		**LS1**		**LS2**		**LS3**		**OS1**		**OS2**		**OS3**	
		Gy-Eq		Gy-Eq (± SD)		Gy-Eq (± SD)		Gy-Eq (± SD)		Gy-Eq		Gy-Eq		Gy-Eq	
		**T**_6.5 **cm**_	**T**_2.5 **cm**_	**T**_6.5 **cm**_	**T**_2.5 **cm**_	**T**_6.5 **cm**_	**T**_2.5 **cm**_	**T**_6.5 **cm**_	**T**_2.5 **cm**_	**T**_6.5 **cm**_	**T**_2.5 **cm**_	**T**_6.5 **cm**_	**T**_2.5 **cm**_	**T**_6.5 **cm**_	**T**_2.5 **cm**_
**NB**	D_mean_	3.82	1.64	3.78 (0.03)	1.62 (0.01)	3.63 (0.04)	1.56 (0.02)	3.42 (0.05)	1.46 (0.02)	3.64	1.56	3.47	1.49	3.30	1.41
	D_max_	12.26	5.25	11.99 (0.16)	5.14 (0.07)	11.70 (0.31)	5.01 (0.13)	11.50 (0.24)	4.93 (0.10)	11.63	4.98	11.17	4.78	10.72	4.59
**CW**	D_mean_	1.83	0.78	1.79 (0.08)	0.77 (0.04)	1.73 (0.20)	0.74 (0.09)	1.63 (0.26)	0.70 (0.11)	1.80	0.77	1.73	0.74	1.62	0.70
**L-ON**	D_mean_	0.85	0.36	0.81 (0.12)	0.35 (0.05)	0.83 (0.15)	0.36 (0.06)	0.84 (0.19)	0.36 (0.08)	0.82	0.35	0.76	0.33	0.68	0.29
**R-ON**	D_mean_	0.92	0.39	0.83 (0.07)	0.36 (0.03)	0.79 (0.14)	0.34 (0.06)	0.79 (0.24)	0.34 (0.10)	0.82	0.35	0.79	0.34	0.73	0.31
**L-lens**	D_mean_	0.29	0.12	0.29 (0.03)	0.12 (0.01)	0.30 (0.05)	0.13 (0.02)	0.29 (0.07)	0.12 (0.03)	0.32	0.14	0.24	0.10	0.21	0.09
**R-lens**	D_mean_	0.32	0.14	0.30 (0.03)	0.13 (0.01)	0.33 (0.10)	0.14 (0.04)	0.29 (0.10)	0.13 (0.04)	0.32	0.14	0.22	0.09	0.22	0.09
**L-eye**	D_mean_	0.37	0.16	0.36 (0.04)	0.15 (0.02)	0.38 (0.07)	0.16 (0.03)	0.37 (0.09)	0.16 (0.04)	0.35	0.15	0.32	0.14	0.29	0.13
**R-eye**	D_mean_	0.39	0.16	0.39 (0.04)	0.16 (0.02)	0.36 (0.08)	0.15 (0.03)	0.35 (0.13)	0.15 (0.05)	0.34	0.15	0.30	0.13	0.28	0.12
**BS**	D_mean_	4.04	1.73	3.86 (0.33)	1.65 (0.14)	3.74 (0.62)	1.60 (0.27)	3.53 (0.87)	1.51 (0.37)	3.75	1.61	3.53	1.51	3.36	1.44

^*∗*^Gy-Eq is the unit of the values reported. NB: normal brain; CW: Circle of Willis; L: left; R: right; ON: optic nerve; BS: brain stem. D: dose; LS1: 1 cm lateral shift; LS2: 2 cm lateral shift; LS3: 3 cm lateral shift; OS1: 1 cm outward shift; OS2: 2 cm outward shift; OS3: 3 cm outward shift. The mean doses reported for LS1, LS2, and LS3 represent the mean values of the doses for left/right and superior/inferior shifts.

## Data Availability

The data used to support the findings of this study are available from the corresponding author upon request.
